# Autofluorescence Lifetime Reports Cartilage Damage in Osteoarthritis

**DOI:** 10.1038/s41598-020-59219-5

**Published:** 2020-02-07

**Authors:** João L. Lagarto, Mohammad B. Nickdel, Douglas J. Kelly, Andrew Price, Jagdeep Nanchahal, Chris Dunsby, Paul French, Yoshifumi Itoh

**Affiliations:** 1 Department of Physics, Imperial College London, London, SW7 2AZ UK; 2 Kennedy Institute of Rheumatology, University of Oxford, Oxford, OX3 7FY UK; 3 Botner Research Centre, University of Oxford, Oxford, OX3 7LD UK; 4 Centre for Pathology, Imperial College London, London, SW7 2AZ UK

**Keywords:** Cartilage, Applied optics

## Abstract

Osteoarthritis (OA) is the most common arthritis and its hallmark is degradation of articular cartilage by proteolytic enzymes leading to loss of joint function. It is challenging to monitor the status of cartilage *in vivo* and this study explores the use of autofluorescence lifetime (AFL) measurements to provide a label-free optical readout of cartilage degradation that could enable earlier detection and evaluation of potential therapies. We previously reported that treatment of *ex vivo* porcine cartilage with proteolytic enzymes resulted in decreased AFL. Here we report changes in AFL of *ex vivo* mouse knee joints, porcine metacarpophalangeal joints, normal human metatarsophalangeal articular tissue and human OA tibial plateau tissues measured with or without treatment using a compact single-point time resolved spectrofluorometer. Our data show that proteolytically damaged areas in porcine metacarpophalangeal joints present a reduced AFL and that inducing aggrecanases in mouse and human joints also significantly reduces AFL. Further, human cartilage from OA patients presents a significantly lower AFL compared to normal human cartilage. Our data suggest that AFL can detect areas of cartilage erosion and may potentially be utilised as a minimally-invasive diagnostic readout for early stage OA in combination with arthroscopy devices.

## Introduction

Osteoarthritis (OA) is the most common joint disease, affecting around 10% of men and 18% of women over 60 years of age^1^. The epidemiology of the disease is complex and multifactorial, with genetic, biological and biochemical components. Traditionally, treatment of OA is centred on pain management, with joint replacement at the end stage of the disease. Despite the clinical success of joint replacement, outcomes in younger patients are less predictable and there is increased interest in disease prevention, early diagnosis^2^ and the development of alternative treatment strategies for early disease. There is therefore interest in identifying diagnostic biomarkers for OA^3^, for which candidates include serum COMP and C-telopeptide of collagen II fragments. However, these biomarkers require biochemical analysis of blood samples and to date there are no established clinical tools to directly detect early stage of OA.

Cartilage is a specialised tissue composed of a dispersed population of chondrocytes and a rich extracellular matrix (ECM). Cartilage ECM is predominantly type II collagen and aggrecan proteoglycan (about 45% each in wet weight)^4,5^. In OA, these components are degraded by proteolytic enzymes that belong to the families of matrix metalloproteinases (MMP) and a disintegrin and a metalloproteinase with thrombospondin motifs (ADAMTS)^6^. Collagen is degraded by collagenases that belongs to MMP and aggrecan is degraded by both MMP and ADAMTS enzymes^6^. It is believed that aggrecan degradation precedes collagen degradation in early OA, and this early aggrecan depletion is thought to be due to aggrecanases belonging to the ADAMTS family of enzymes including ADAMTS4 and 5^6^. Indeed, ADAMTS5 null mice were protected from cartilage degradation in an experimental OA model^7^. This suggests that aggrecan removal is a crucial early event in OA progression and therefore a minimally invasive tool that could report aggrecan depletion would be of great interest for early diagnosis of OA.

Autofluorescence, which is the optically excited emission from endogenous fluorescent molecules in biological tissue, has previously been explored as a label-free means to report on cartilage degradation using spectrally resolved measurements^8–10^. Further information can be gained from autofluorescence lifetime (AFL) measurements of cartilage. The fluorescence lifetime is the average time during which an excited electron remains in the excited state and is a spectroscopic parameter that can distinguish different molecular species or variations in the local chemical or physical environment^11^. Since fluorescence lifetime measurements of a single fluorescent species are independent of the efficiency of excitation and detection of the emission, they can be more robust than fluorescence intensity measurements, particularly in complex scattering samples such as biological tissue^12^.

The application of fluorescence lifetime readouts to cartilage autofluorescence was reported in 2004^13^ and subsequent work on *ex vivo* engineered cartilage tissue has shown the potential for autofluorescence lifetime (AFL) measurements to provide label-free readouts of cartilage degradation following specific treatments with collagenase, chondroitinase-ABC and ribose^14^. We have previously reported that both the degradation of collagen and the removal of aggrecans from natural *ex vivo* cartilage are associated with a decrease in AFL of cartilage tissue following treatment with bacterial collagenase, human MMP-1 and, trypsin; and also aggrecanase induction by retinoic acid treatment of live cartilage^15^.

Collagen is a major contributor of cartilage autofluorescence through its intermolecular crosslinks^16,17^. As the gaps between collagen fibrils are largely filled by aggrecans in cartilage tissue^4,5^, removal of aggrecan is likely to influence the chemical microenvironment of collagen crosslinks. This may explain why proteolytic removal of aggrecan from cartilage can reduce the AFL of cartilage collagen^15^. Here we extend our previous work to evaluate the potential of AFL measurements to provide label-free readout of cartilage damage with a view to the development of a clinical instrument for the non-invasive monitoring of cartilage status. Specifically, this study focused on (1) how chemically-induced aggrecan depletion affects the AFL of intact murine, porcine and human cartilage; (2) whether AFL measurements can report local enzymatically-induced damage in the articular cartilage surface; (3) determining if AFL measurements can be used to detect natural erosion in human tibial plateau cartilage.

## Methods

### Materials

Bovine trypsin and retinoic acid were purchased from Sigma-Aldrich (Dorset, UK). Bacterial collagenase type 2 was purchased from Worthington Biochemical Corp (Lakewood, NJ, USA). Recombinant MMP-1 was expressed in E.coli, purified and activated as previously reported^18,19^.

### Tissue treatment

Mouse knee joints were obtained from 8 weeks old C57 BL/6 male mice. The knee joints were used for experiments immediately after dissection. Whole murine knee joints were treated with active matrix metalloproteinase-1 (MMP-1, 50 μg/ml) at 37 °C for 24 hours.

Porcine metacarpophalangeal joints were obtained from slaughterhouse. The joint tissue was used for experiments immediately after dissection. Local degradation of porcine cartilage was induced by placing a 5 mm diameter disc of filter paper soaked with 20 μl of bacterial collagenase (500 μg/ml), MMP-1 (500 μg/ml) or trypsin (200 μg/ml) solution on the surface of cartilage and incubating it in a humidified 37 °C cell culture incubator for 24 hours.

All the protocols using animal tissue (mouse and porcine) were approved by the University of Oxford and carried out in accordance with the regulation and the guidelines of University of Oxford.

Normal fresh human metatarsophalangeal articular tissue was obtained following informed consent from patients (n = 4 patients) undergoing amputation after approval by the NRES committee London Riverside (IRAS ID: 46584) under REC reference 07/H0706/81. The fresh joint tissues were treated with 10 μM retinoic acid (RA) in culture for 72 hours to induce aggrecan release. All the experimental protocols and methods using human tissue have been carried out in accordance with the regulation and the guidelines of University of Oxford.

### Human OA joint tissue

Human OA tibial plateau tissues (n = 2 patients) were obtained from the Oxford Musculoskeletal Biobank (09/H0606/11) and were collected from patients undergoing uni-compartmental knee arthroplasty with informed donor consent in full compliance with national and institutional ethical requirements, the United Kingdom Human Tissue Act, and the Declaration of Helsinki. These OA specimens had a Kellgren and Lawrence **(**KL**)** grading of IV. All OA tissue samples were kept at −80 °C until AFL measurements. All experimental protocols using human OA tissue were carried out in accordance with the University of Oxford regulation and the guidelines.

### Bench-top single-point fibre optic multidimensional fluorometer probe

AFL measurements from murine and porcine samples were realised using a bench-top single-point multidimensional fluorometer as previously described^15,20,21^. This instrument provides spectral and time-resolved information of the fluorescence signal using an optical fibre probe extension, which facilitates measurements from tissue samples. Excitation light at 5 MHz was provided by a 375 nm laser diode (LDH-P-C-375B, PicoQuant GmbH, Germany) generating ~70 ps optical pulses. Excitation light was delivered to the sample via a custom-built fibre optic probe (NA = 0.22, FiberTech Optica, Canada) incorporating three multimode excitation fibres and sixteen multimode collection fibres. At the proximal end, fluorescence light emanating from the sample was focused onto the input slit of a motorised monochromator (CM110, CVI Inc., USA) before being directed to a cooled photomultiplier tube (PMT, PMC-100-1, Becker & Hickl GmbH, Germany) to provide single channel, spectrally resolved detection with time-resolution implemented using time-correlated single photon counting (TCSPC, SPC-730, Becker & Hickl GmbH, Germany). Autofluorescence from the specimens excited at 375 nm was measured at the emission peak wavelength, i.e. 460 nm (data not shown).

### Single-point time-resolved spectrofluorometer

AFL measurements of human samples were realised using a more compact fibre optic based time-resolved spectrofluorometer previously described elsewhere^22^. The instrument comprises two excitation sources, although for the measurements reported below we only used a 375 nm laser diode (LDH-P-C-375B, PicoQuant GmbH, Germany) that provides ~70 ps optical pulses. The average power at the sample plane was kept below 25 μW. The laser repetition rate was adjusted to 5 MHz. Excitation light was delivered to the sample via a bifurcated optical fibre bundle (NA = 0.22, FiberTech Optica, Canada) that consisted of three multimode excitation fibres and fourteen multimode detection fibres. Fluorescence from the sample was delivered to the detection arm that consisted of a set of filters and dichroic mirrors that separate the signal in three bands to provide spectral discrimination: 400–420 nm (channel 1); 430–480 nm (channel 2); 500–550 nm (channel 3). Each channel has a photon-counting PMT (PMC-100–1, Becker & Hickl GmbH, Germany) connected to a TCSPC card (SPC-830, Becker & Hickl GmbH, Germany) via a router (HRT-41, Becker & Hickl GmbH, Germany). The TCSPC system records the temporal fluorescence intensity decay profile for each spectral channel.

### Data analysis

The fitting of fluorescence lifetime data was undertaken using a custom software application in MATLAB. All data were fitted to a double exponential decay model to parameterize the fluorescence decay characteristics. The intensity weighted mean autofluorescence lifetime was calculated as

1 $${\tau }_{{\rm{mean}}}=\frac{{a}_{1}{\tau }_{1}^{2}+{a}_{2}{\tau }_{2}^{2}}{{a}_{1}{\tau }_{1}+{a}_{2}{\tau }_{2}}$$

where *a*_1_, *a*_2_ and *τ*_1_, *τ*_2_ are the pre-exponential factors and fluorescence lifetimes of the fast and slow components of the fluorescence intensity decay, respectively.

### Statistical analysis

GraphPad Prism was used for statistical analysis and data visualisation. All data were expressed as mean ± standard deviation (SD). Statistical analyses of different groups were analysed by Student’s t-test. Each experiment’s data sizes are described in the figure legend. P-values for each experiment are stated in the figure legends.

## Results

### Autofluorescence lifetime reports degradation of animal cartilage

**Figure 1:**
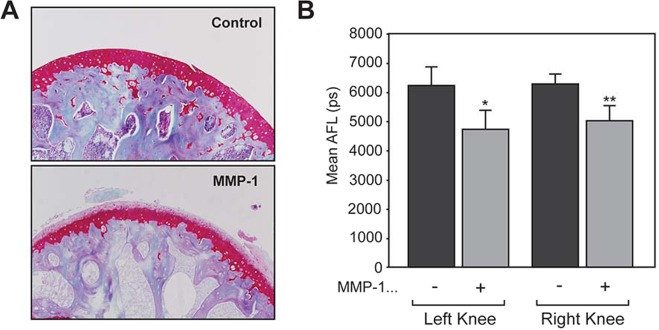
Effect of MMP-1 on AFL of mouse knee cartilage. Fresh mouse knee joints (n = 5) were incubated in the buffer containing with 50 µg/ml recombinant active human MMP-1 at 37 °C for 24 hours. (**A**) Tissues treated with MMP-1 were analysed histochemically in comparison to the control tissues without MMP-1 treatment as indicated. Samples were stained with Safranin-O to stain proteoglycans in cartilage tissue (stained red) against counter staining of haematoxilin (purple). The data are representative images from each group. (**B**) Treated tissues were subjected to measurement of autofluorescence lifetime (AFL). The graph indicates the mean autofluorescence lifetime (mean AFL).*p > 0.05, **p > 0.01.

We previously reported that treatment of porcine cartilage pieces with proteolytic enzymes decreased autofluorescence lifetime (AFL) of the tissue^15^. To examine if proteolytic damage of the cartilage can be detected directly on the joint, whole murine knee joints were treated with active matrix metalloproteinase-1 and the AFL was measured using the benchtop instrument^15^ to detect autofluorescence at 460 nm. MMP-1 is a collagenase that degrades cartilage collagen. As shown in Fig. 1A, mouse knee joints treated with MMP-1 showed depletion of aggrecan from cartilage across the surface. This is consistent with our previous work on bovine cartilage^15^. The AFL of non-treated murine knee joints was 6.2 ± 0.6 ns and treatment with MMP-1 resulted in a statistically significant decrease to 4.9 ± 0.7 ns (p < 0.01, n = 10, Fig. 1B). Although we are not able to conclude if this decrease is due to aggrecan depletion or collagen degradation, the data suggest that proteolytic damage caused by MMP-1 can be detected by AFL in mouse joint cartilage.

**Figure 2:**
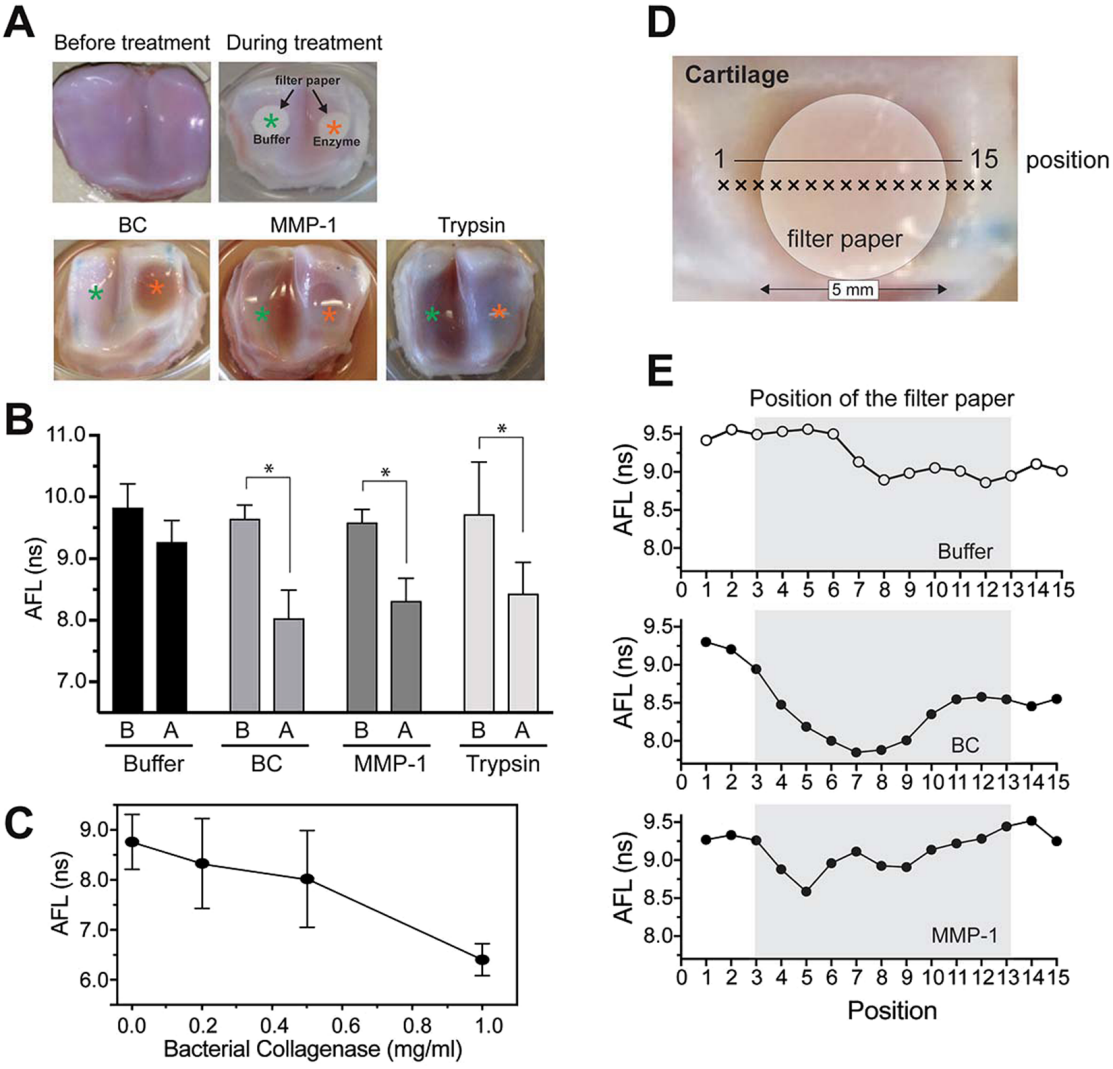
Detection of local proteolytic damage by AFL measurement. **(A**) Representative photos of cartilage samples before and after treatment. Cartilage surface of porcine metacarpophalangeal joints were treated with bacterial collagenase (500 μg/ml), MMP-1 (500 μg/ml) and trypsin (200 μg/ml) by placing filter papers soaked with these proteinase solutions. Samples were incubated at 37 °C for 24 hours with enzymes or buffer prior to measurement in a humidified cell culture incubator to prevent drying of cartilage samples. (**B**) Mean autofluorescence lifetime of articular cartilage samples after treatment with bacterial collagenase, MMP-1 and trypsin. The data indicate that treatment of porcine cartilage with these enzymes decreased AFL in a statistically significant manner: bacterial collagenase, p < 0.0001; MMP-1, p < 0.0004; and trypsin, p < 0.0072 (n = 9 for each group). (**C**) Porcine joint cartilage was treated with increasing dose of Bacterial collagenase as indicated, and mean AFL was measured (n = 6). (**D**) Location of line profile measurements along the cartilage surface. (**E**) Mean autofluorescence lifetime along the cartilage surface treated with buffer (top), bacterial collagenase (middle) and MMP-1 (bottom).

Using porcine metacarpophalangeal joints, we next examined if local damage of cartilage can be detected by AFL measurements at 460 nm using the benchtop instrument^15^. Local degradation of the cartilage was induced by placing a 5 mm diameter disc of filter paper soaked with bacterial collagenase, MMP-1 or trypsin on the surface of cartilage and incubating it at 37 °C for 24 hours. Bacterial collagenase is a strong proteinase that degrades collagen and aggrecans rapidly, while trypsin does not degrade collagen, but aggrecan. AFL measurements were realised in areas where the buffer-soaked or enzyme-soaked filter papers were placed. As shown in Fig. 2, areas of the cartilage surface treated with bacterial collagenase (BC)(p < 0.0001, n = 9), MMP-1 (p < 0.001, n = 9), or trypsin (p < 0.01, n = 9) showed a significantly shorter AFL relative to untreated control regions (Fig. 2B). Since trypsin treatment decreased AFL in a similar level to BC and MMP-1 treatment, this decrease may be mainly attributed to aggrecan loss. We note that treatment with buffer alone also seems to induce a decrease in the AFL, although on a smaller scale compared to enzymatically treated regions, which may be attributed to the release of proteoglycans from tissue without proteolytic degradation, potentially leading to changes in the mechanical properties of cartilage that may affect cartilage AFL^23^. We have also observed a decrease in AFL that correlates with increase in enzyme concentration (Fig. 2C), which suggests that the decrease of AFL is indeed due to proteolytic action. To investigate whether AFL measurements can report local cartilage degradation, line profile measurements were realised by scanning the fibre probe along the cartilage surface as shown in Fig. 2D. The results (Fig. 2E) indicate a decrease in AFL in the eroded area in bacterial collagenase-treated samples. MMP-1 treated cartilage showed a smaller decrease for part of the treated area. These results suggest that AFL may be used to map areas of cartilage degradation.

### AFL reports degradation of human cartilage

**Figure 3:**
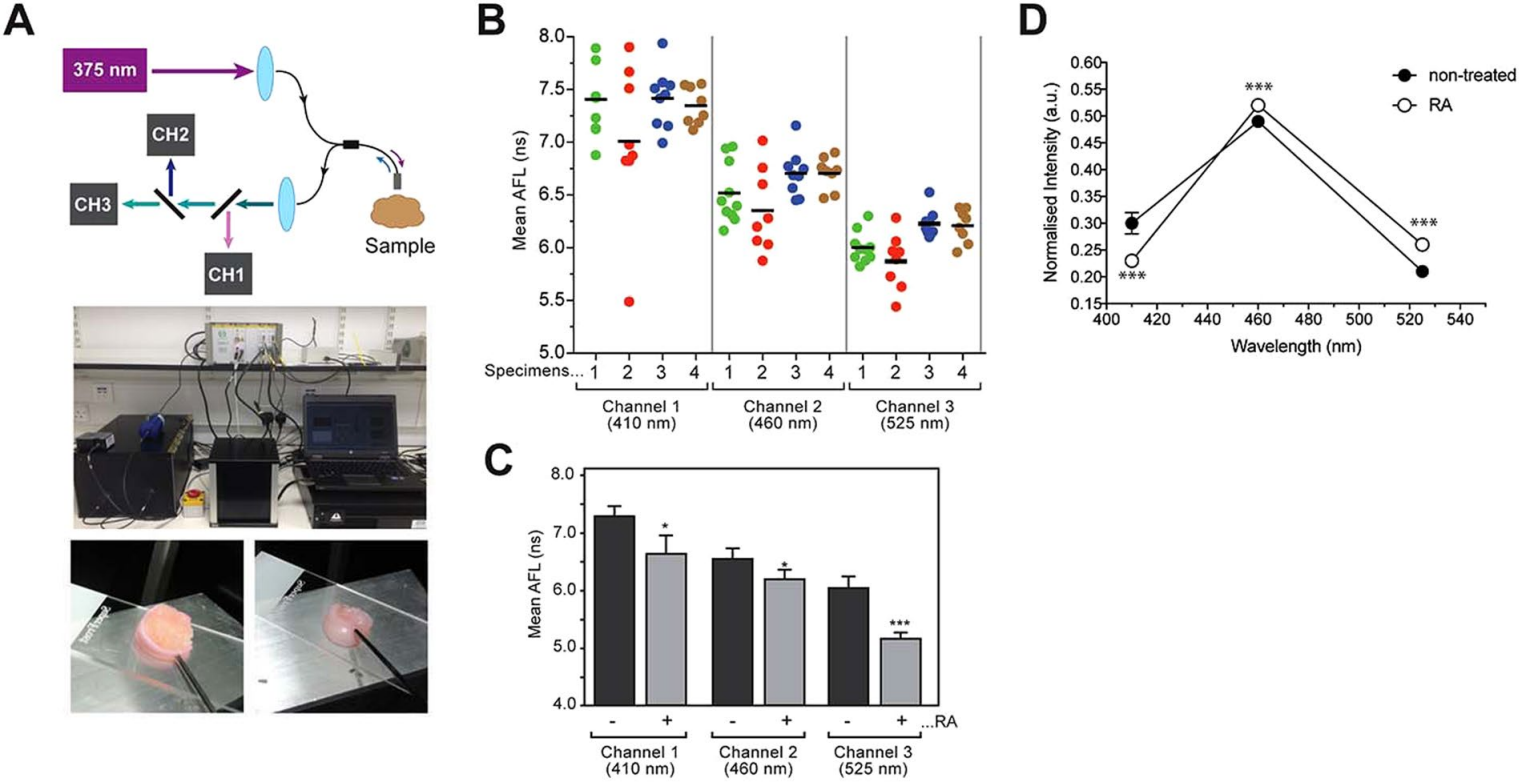
AFL measurement of human cartilage. (**A**) At the top, a photograph of the single-point time-resolved spectrofluorometer deployed in this experiment is shown. At the bottom, representative photos of human metatarsophalangeal articular cartilage specimens are shown with the fibre optic point probe in contact with the tissue. (**B**) Intra- and inter-sample variability of AFL in untreated “normal” specimens. Human metatarsophalangeal articular cartilage specimens were subjected to AFL measurement at different positions in each specimen (6–9 positions/measurements). Different colours identify different specimens. Horizontal bars indicate the average weighted mean fluorescence lifetime over all specimens. (**C**) Fresh human metatarsophalangeal articular cartilage was treated with or without retinoic acid for 7 days in the culture medium followed by AFL measurement. AFL significantly decreased with retinoic acid treatment. p < 0.0127*, p < 0.0280* and p < 0.0005*** were calculated for channels 1, 2 and 3, respectively (n = 4). (**D**) Relative contribution of each spectral channel to the overall fluorescence intensity at 375 nm excitation. Each individual point is the average over 4 specimens. The standard deviation was also calculated for each point and its average over all points on the graph is indicated by the error bars on the black circle on the left-hand side. ***p < 0.0005, p < 0.0056 and p < 0.0008 were calculated for channels 1, 2 and 3, respectively (n = 4).

To investigate the autofluorescence lifetime signatures of human articular cartilage, we used the compact single-point time-resolved spectrofluorometer employing a fibre-optic probe to deliver UV excitation light and collect autofluorescence signal (Fig. 3A top) with the fluorescence decay being recorded in three detection channels centred at 410, 455 and 525 nm. Using this instrument, we measured the AFL of normal human metatarsophalangeal articular cartilage (Fig. 3A bottom). We noted some intra- and inter-sample variation of AFL that may be due to natural variations in the tissue (Fig. 3B). If we take the average lifetime of each specimen at each channel (i.e. n = 4 specimens), we obtained mean autofluorescence lifetimes of 7.3 ± 0.2 ns, 6.6 ± 0.2 ns and 6.1 ± 0.2 ns for channels 1, 2 and 3, respectively (Fig. 3B). These results suggest that AFL of normal healthy human cartilage is wavelength dependent for this excitation at 375 nm.

Next, fresh normal human metatarsophalangeal articular joints were treated with retinoic acid (RA) followed by AFL measurements. Treating live cartilage with RA stimulates aggrecanase production and removes aggrecan without collagen loss^15^. As shown in Fig. 3C, treatment of joint tissues with RA reduced AFL in a significant manner in all detection channels. We also observed statistically significant changes in the fluorescence emission spectra of treated samples (Fig. 3D).

### Measurement of AFL on human OA cartilage

**Figure 4:**
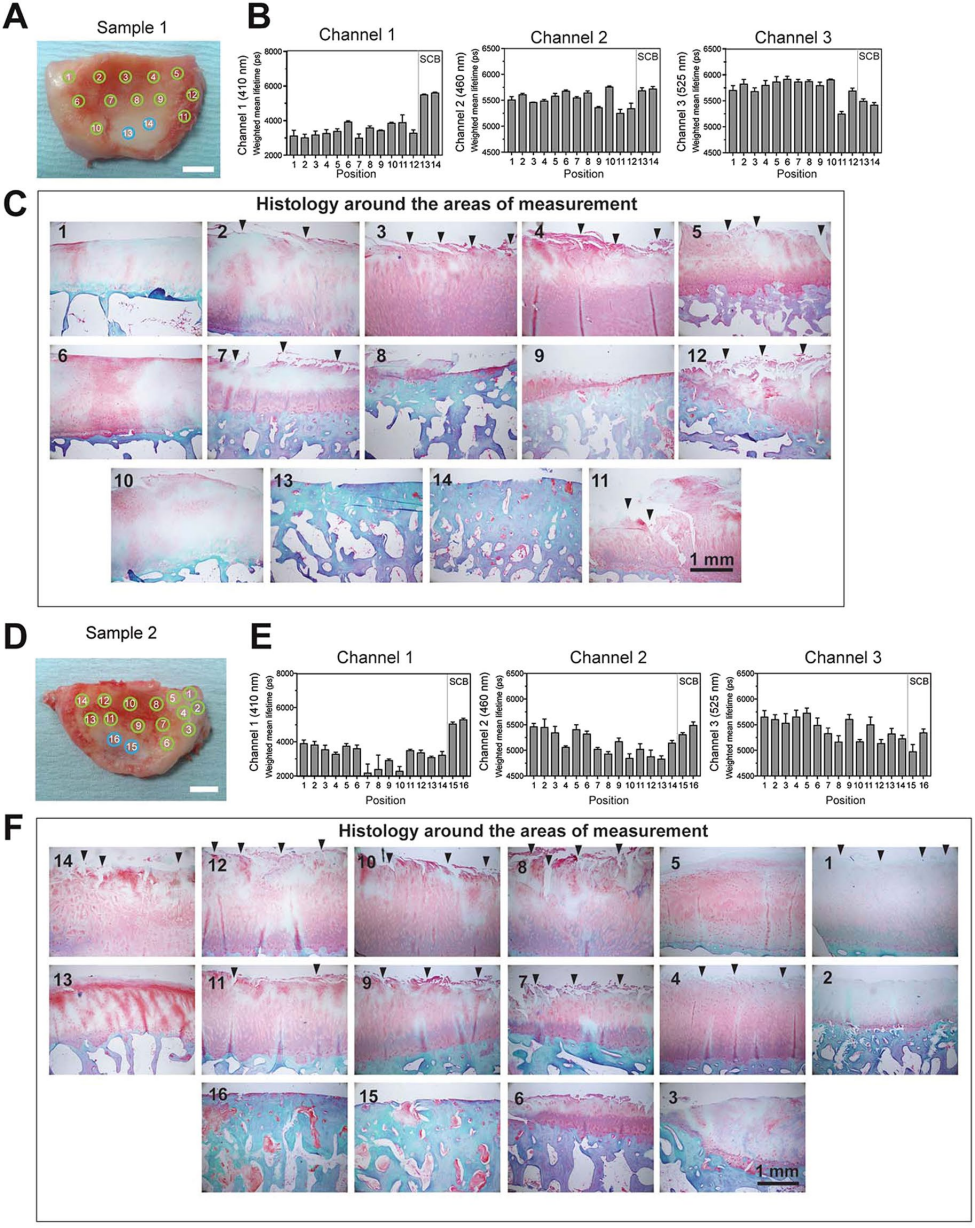
AFL measurement of human OA cartilage. Analyses from two OA cartilage samples are shown (sample 1 and 2). These OA specimens had a KL grading of IV. Top left image shows photos of sample with the approximate position of the fibre optic probe at the time of measurements, indicated by the green (visibly remaining cartilage) and blue circles (exposed bone). Scale bar 1 cm. AFL results in each spectral channel 1, 2 and 3 are presented below. A total of n = 3 AFL measurements were realized in each position. The tissues were sliced into 9 strips and histological sections were made. Histological images of the closest position from the measurement area of the tissue were shown in the right. Arrowheads indicate tears on the cartilage surface. Scale bar 1 mm. The sections were stained with safranin O and Masson’s trichrome. SCB indicates regions of exposed subchondral bone.

All results so far demonstrated that AFL can report cartilage degradation upon enzymatic treatment, which suggests that AFL measurements may help diagnosis of OA. We thus next analysed AFL of human OA cartilage tissues. Human tibial plateau tissue specimens were obtained with consent from material removed during uni-compartmental knee arthroplasty and analysed by single-point time-resolved spectrofluorometry in multiple locations on the tissue to cover areas of cartilage in different conditions, as shown in Fig. 4. The tissues were then fixed and embedded in the paraffin to inspect the microscopic tissue damage. Tissue were stained with safranin O for aggrecan and Masson’s trichrome for collagen staining. AFL measurements were realised in areas where cartilage remained yet was visibly degraded (green circles) and areas with exposed subchondral bone (SCB, blue circles). The data from sample 1 indicate that AFL values of OA cartilage are notably shorter (3.4 ns for channel 1, 5.5 ns for channel 2, and 5.6 ns for channel 3) than that from healthy metatarsophalangeal cartilage data with average of 7.3 ns for channel 1, 6.7 ns for channel 2, and 6.3 ns for channel 3 (Fig. 3). Among the three channels, channel 1 shows the largest shift in OA tissue compared to healthy tissue. At positions 2–6 in Sample 1, the cartilage is visibly thicker compared to other positions – as seen by its redder appearance in Fig. 4A and by the pink/white regions in the histology – but AFL values are as low as other areas. Histological analyses of the tissue around the measured positions indicate that there are notable tears of the cartilage surface (positions 2, 3, 4, 5, 7, 11, 12, arrowheads) and loss of aggrecans as indicated by weak and uneven red safranin O staining (all positions), so the reduced AFL is consistent with chemical environmental changes that cannot be seen by visual inspection of the tissue. However, these AFL measurements cannot distinguish the nature of cartilage damage between tears (collagen degradation) and aggrecan loss. Exposed bone areas yield lifetimes of 5.4 and 5.6 ns (channel 1, position 13 and 14).

For sample 2 the AFL values are also much lower than that of healthy cartilage (Fig. 3), especially for channel 1. Histology data indicate that cartilage has extensive tears (position 1, 4, 7, 8, 9,10, 11, 12, 14 arrowheads), and loss of aggrecans (all positions). Exposed bone area (position 15 and 16) gave lifetimes of 5.2 and 5.4 ns (channel 1), which is consistent with sample 1. These data strongly indicate that AFL can indeed report cartilage damage in human OA.

## Discussion

Cartilage is a vital organ to maintain joint function, and in arthritic joints, such as in patients with osteoarthritis or rheumatoid arthritis, its function is compromised due to proteolytic degradation of cartilage matrix components including aggrecan and collagen^6^. At present, the only way to detect these molecular events in cartilage tissue *ex vivo* is physical dissection of the cartilage followed by histological analysis. The standard methodologies available to monitor the condition of cartilage *in vivo* and aid OA diagnosis are x-ray or magnetic resonance imaging (MRI). Although these techniques can provide relevant structural information of the tissue, alterations in cartilage morphology typically occur in the mid to late stages of disease progression, when cartilage has already been deformed and eroded.

We have previously reported that AFL can report proteolytic damage of *ex vivo* cartilage tissue that includes aggrecan degradation in porcine cartilage pieces^15^. Similar work by others demonstrated that AFL can report glycosaminoglycan (GAG) depletion in bovine articular cartilage^24^. In this study, we investigated the potential of AFL to report areas of proteolytically damaged human cartilage. Partial treatment of cartilage surface with bacterial collagenase, human interstitial collagenase, MMP-1, and trypsin resulted in significantly lower AFL values compared to areas treated with buffer alone or before treatment. Point measurements along a line through the treated area indicated that AFL has the potential to map the area with proteolytic damage (see Fig. 2D,E). Since trypsin degrades aggrecan but not collagen, the apparent shape of cartilage in this area was maintained with trypsin treatment. Thus, AFL measurements have the potential to detect early cartilage defects with aggrecan removal that cannot be detected by conventional diagnostic methods. In addition to clinical applications, AFL may also be able to contribute to analyses of cartilage degradation in pre-clinical animal models of OA for the development of potential therapeutics. The current size of the fibre optic probe is a diameter of around 2 mm, and it can be used for a *in vivo* disease model using dog and horse, although it is too large for a *in vivo* measurement in mouse, a common species used for OA disease model. In the future, a smaller and more sophisticated fibre-probe tip could be developed, which would permit measurements in smaller animals including mouse.

In general, our results indicate that healthy cartilage yields a complex fluorescence decay profile, with average lifetimes ranging from 5–6 ns, 9–10 ns, and 7–8 ns (channel 1) in murine, porcine and human cartilage, respectively. We attribute these autofluorescence lifetimes, and corresponding wavelength dependency in the case of human samples, to the presence of collagen type II and a range of crosslinks and associated fluorophore environments. Previous studies have demonstrated that fluorescence characteristics of commercially available purified collagen are highly dependent on the source of collagen^12,25,26^. It is well known that cartilage composition varies with species and mechanical load, which translates into different collagen arrangement, proteoglycan concentration and thickness^27,28^, which may result in different AFL for different species. For example, our observation of a wavelength dependence of the AFL of human cartilage was not observed in our previous measurements of digested porcine cartilage explants^15^ excited at 355 nm and may be attributed to different crosslinking fluorophore environments found in human samples compared to porcine samples. In the future, biochemical studies will be undertaken to quantitatively measure collagen crosslinks from human samples to further understand the origins of the fluorescence signal.

Inducing proteolytic damage in cartilage tissue resulted in significant decrease in AFL in murine, porcine and human joints. These measurements confirmed previous observations^15,24^, demonstrating that AFL is sensitive to biochemical alterations in the cartilage ECM caused by disruption of collagen crosslinks or aggrecan depletion from tissue. In particular, we observed a decrease in the AFL of fresh human metatarsophalangeal articular cartilage treated with RA. Treatment with RA promotes production of aggrecanases in chondrocytes leading to aggrecanolysis. This result suggests that AFL can report aggrecan loss in human cartilage, which is a relevant step towards early detection of OA.

We have analysed two specimens of human OA cartilage from the tibial plateau. These samples were from end stage OA, and thus bone is exposed in some areas. We noted that the AFL of the OA cartilage was around 2.2–3.8 ns in channel 1, which is significantly lower than the typical values obtained with healthy human metatarsophalangeal articular cartilage, suggesting that AFL is indeed reporting cartilage damage. Unfortunately, healthy normal tibial plateau cartilage tissues were not available for direct comparison, thus it is not clear how much decrease in AFL occurred upon cartilage erosion and how variable AFL values are between different individuals and between different joints. This limitation of our study arises because “normal” healthy human cartilage tissue is usually not available *ex vivo*. Future work will focus on *in vivo* measurements of healthy and disease cartilage tissues of the same joint. In particular, we will investigate whether AFL measurements can report changes between medial and lateral regions of the joint. Given that medial regions are affected first in OA, differences in the AFL of medial and lateral sides could provide an early indicator of the disease.

Changes in the AFL signature of human OA cartilage are challenging to interpret. Our previous results for enzymatically digested cartilage indicated that the AFL of cartilage decreases proportionately with level of degradation in all detection channels relative to untreated samples^15^. However, cartilage degradation in OA cartilage is a mixture of collagen degradation and aggrecan degradation, and AFL cannot distinguish them – even when measuring AFL in the three spectral channels. Overall, our results suggest that channel 1 provides the strongest correlation with general cartilage wear, including both aggrecan removal and/or collagen degradation. Comparing the shift of AFL signature between enzyme treatment and OA damage, enzyme treatment resulted in larger decrease of AFL in channel 3 compared to channels 1 and 2, while in OA cartilage the fluorescence lifetime was shortest in channel 1 compared to channels 2 and 3. It is not clear why treating cartilage with proteolytic enzymes did not induce more prominent decrease in the channel 1 relative to channel 2 or 3, but it may suggest that the *ex vivo* enzyme treatments are not fully mimicking the disease state of the cartilage. In the present study it was not possible to measure the AFL signatures of normal healthy human tibial plateau cartilage tissues; thus, it remains unclear whether channel 1 lifetime is more affected than channel 3 in OA samples, with respect to normal/healthy tissues. The spread in AFL values in channel 1 from 4 patients in Fig. 3B is as tight as 7.3 ± 0.2 ns (mean ± standard deviation), indicating that this may be a typical AFL value of normal healthy human cartilage. This AFL is well separated from the values of OA tissue (<4 ns) observed in Fig. 4, suggesting that it may be possible to classify normal/OA patients based on the measured channel 1 lifetime without a “normal” control measurement. However, this would need to be further investigated in a larger scale study in the future.

In this study, we employed 375 nm light to excite autofluorescence from cartilage tissues. The penetration depth of 375 nm light is limited, typically ranging from 100 to 200 μm, depending on the scattering and absorption properties of the tissue. Considering that collagen is highly scattering, we believe that most of our AFL signal emanates from the top 100 μm layer and is unlikely to be affected by changes in deeper layers of the tissue. Thus, in human and porcine samples where cartilage thickness is ~2 mm^27^, AFL reports changes in tissue occurring in the exposed surface of cartilage, which can correspond to any layer of the cartilage depending on the stage of the degradation. On the other hand, since murine cartilage is much thinner (~100 μm)^27^, the autofluorescence likely originates from the various layers and the measured AFL signals reflect overall changes in cartilage.

The optical fibre-based single-point AFL instrument is sufficiently compact and portable to be deployed in clinical environments. This approach could be adapted for use with existing arthroscopes to measure the autofluorescence signal of cartilage *in vivo* in patients. We believe that AFL measurements have potential to aid OA diagnosis, particularly in early stages of development of the disease when current modalities cannot provide useful diagnostic information. Another advantage relative to standard OA diagnostic methods is the relatively low cost of the AFL instrumentation compared to MRI or x-ray scanners. We note that the prototypes used here have a component cost of ~£40,000–50,000 and that we have shown this can be significantly reduced using low-cost custom electronics^21^.

In summary, the work presented in this paper demonstrates that time-resolved autofluorescence spectroscopy may offer a minimally-invasive and label-free readout of cartilage matrix integrity that could contribute to future diagnosis of early cartilage defects as well as monitoring the efficacy of therapeutic agents. Further investigations are still necessary to compare normal and OA human cartilage and these will probably need to be undertaken *in vivo*, e.g. using modified arthroscopes.
